# A Q fever outbreak among visitors to a natural cave, Bizkaia, Spain, December 2020 to October 2021

**DOI:** 10.2807/1560-7917.ES.2023.28.28.2200824

**Published:** 2023-07-13

**Authors:** Ana Hurtado, Ion I Zendoia, Eva Alonso, Xabier Beraza, Joseba Bidaurrazaga, Blanca Ocabo, Iñaki Arrazola, Aitor Cevidanes, Jesús F Barandika, Ana L García-Pérez

**Affiliations:** 1 Animal Health Department, NEIKER-Basque Institute for Agricultural Research and Development, Basque Research and Technology Alliance (BRTA), Derio, Bizkaia, Spain; 2 Departamento de Salud del Gobierno Vasco, Subdirección de Salud Pública de Bizkaia, Servicio de Epidemiologia, Bilbao, Bizkaia, Spain; 3 Servicio de Ganadería, Departamento de Agricultura, Diputación Foral de Bizkaia, Bilbao, Bizkaia, Spain; * These authors contributed equally to the work and share first authorship.

**Keywords:** Q fever, *Coxiella burnetii*, outbreak, natural caves, small ruminants, genotyping, viability

## Abstract

We describe a large Q fever outbreak reported in Spain, including 108 cases, 53 with pneumonia and 27 requiring hospitalisations. The first cases were detected in February 2021 among rock climbers visiting a cave in Bizkaia, and the last case was detected in October 2021. Most cases were notified after the Easter holiday (April–May 2021). More males (63.9%) than females (36.1%) were infected (median ages: 42 (1–68) and 39 years (6–61), respectively). We detected *Coxiella burnetii* by PCR in faecal, dust and/or aerosol samples taken inside the cave in March 2021, and in dust and aerosol samples collected between March 2021 and February 2023. *Coxiella burnetii* from dust samples were cultured on Vero cells, showing viability for 24 months. Based on serological and genotyping data, goats sheltering in the cave were the most likely source of infection. The cave was closed on 29 April 2021, movements of goats and sheep in the area were restricted (March–July 2021), and the animals were vaccinated in October 2021. Investigation of Q fever outbreaks requires a multidisciplinary One Health approach as these outbreaks can occur in unexpected places like natural sites where animals are present.

**Key public health message**

**What did you want to address in this study?**

Q fever is a zoonotic disease caused by *Coxiella burnetii*, a bacterium highly resistant to environmental stress. The most common reservoirs are sheep, goats and cattle. We investigated a large Q fever outbreak in Spain, which affected over 100 visitors to a natural cave. The clinical characteristics, the infection source, and the persistence of *C. burnetii* in this natural environment were investigated.

**What have we learnt from this study?**

Q fever outbreaks can occur in unexpected places, such as natural sites for sport or recreation where animals are commonly present. The temperature and humidity conditions inside the cave, used as an animal shelter during the lambing/kidding season, contributed to the survival of *C. burnetii* in the environment for 24 months. The closure of the cave to people and animals helped to stop the outbreak.

**What are the implications of your findings for public health?**

Zoonotic infections may occur not only through direct animal contact but also via the environment. To contain outbreaks, recommendations from the public health experts should be followed, and cleaning and disinfection staff should use personal protective equipment. Reducing the prevalence of *C. burnetii* in domestic livestock will decrease the risk of outbreaks.

## Background

Q fever is a zoonotic disease caused by *Coxiella burnetii*, a bacterium highly resistant to environmental stress [1]. The most common reservoirs are ruminants, primarily sheep, goats and cattle. Humans typically acquire Q fever by inhaling aerosols contaminated with *C. burnetii* shed by infected animals. Although most infections with *C. burnetii* are asymptomatic, acute infections may present as influenza-like illness, hepatitis, pneumonia, myocarditis or pericarditis [2]. Outbreaks in humans often affect people with certain occupations such as farmers, veterinarians and slaughterhouse workers. However, long-distance dispersion of *C. burnetii* through the wind [3,4] can also lead to clusters of cases, as seen in many community-based outbreaks reported in several countries [4-10].

In 2019, the incidence of Q fever in the European Union/European Economic Area (EU/EEA) was 0.2 cases per 100,000 inhabitants [11], with a total of 958 confirmed cases. In Spain, the incidence in 2019 was the highest in Europe (0.7/100,000 inhabitants) and, of the different Spanish regions, the Basque Country has historically reported the most cases and the highest number of recorded outbreaks [12]. The first outbreaks were reported in the early 1980s [13,14]. Since then, several Q fever outbreaks involving goats and occasionally sheep have been investigated and were mostly linked to a lack of farm biosecurity measures [15]. The most common genotypes in the investigated outbreaks based on single nucleotide polymorphism (SNP) analysis and multispacer sequence typing (MST) were SNP8/MST18 and SNP1/MST13 [15].

### Outbreak detection

At the end of February 2021, a Q fever outbreak was suspected when several rock climbers reported pneumonia and fever after visiting a cave located within the boundaries of a natural park in Bizkaia, Basque Country, Spain. On 3 March 2021, upon declaration of a Q fever outbreak that affected six rock climbers, a multidisciplinary team consisting of microbiologists, veterinarians, occupational health technicians and epidemiologists gathered to investigate the infection source, monitor cases and plan control measures. On 4 March, *C. burnetii* DNA was detected in preliminary analyses of faecal, dust, and aerosol samples collected inside the cave. In April, several new cases were notified among visitors after the Easter holidays, before the cave was closed to the public on 29 April.

We describe the investigation and control measures implemented in a large outbreak of Q fever in Spain, linked to sport/tourism activities in a natural environment. The outbreak occurred during the period of confinement in the region because of the COVID-19 pandemic, which imposed severe restrictions to people’s mobility in cities and towns.

## Methods

### Outbreak setting

**Figure 1:**
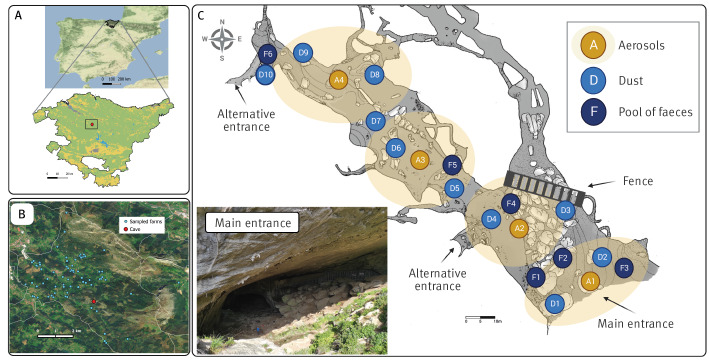
Illustration of the natural cave linked to the Q fever outbreak, Bizkaia, Basque Country, Spain, December 2020–October 2021

The limestone cave, located in Bizkaia, the Basque Country of Spain, is visited annually by tourists and outdoor enthusiasts, including rock climbers. Bat colonies inhabit the lateral gallery of the cave, which is closed to public with a metal fence (Figure 1). A panel next to the fence with information on bat populations is a popular spot among visitors.

A. Map of Spain with an inset showing the location of the cave (red circle).

B. Location of the goat and sheep farms sampled and tested (blue dots) in relation to the cave (red circle).

C. Topographic map of the cave (developed by Jabier Les, G.E.T. Espeleologi taldea). Sampling sites of faecal (F1–F6), dust (D1–D10) and aerosol (A1–A4) samples are indicated. Yellow circular shadows represent the area covered by the air sampler. The approximate location of the fence that prevents entrance to the area with bats is indicated (using BioRender, https://www.biorender.com).

In the municipality of the cave, there are approximately 81 small-scale non-professional farms (< 100 animals/farm) with goats and sheep, and four larger farms (> 100 animals/farm) managed by professional farmers for milk, cheese or meat production. These animals graze outdoors most of the year and some have access to the outer sections of the cave.

### Epidemiological investigation of human cases

Q fever is a compulsorily notifiable communicable disease in Spain; data collection and analysis are performed within the Basque epidemiological surveillance system and submitted to the Spanish Microbiological Information System. To increase awareness of the outbreak and identify as many cases as possible, informative notes were distributed among the local healthcare network, to members of the Basque Mountain Federation, and to the public. All suspected cases received a questionnaire, as detailed in the Q fever surveillance protocol of the Basque Country. An epidemiologist from the Basque Epidemiological Surveillance Unit contacted the suspected cases by telephone and collected information on the following: personal data (age, sex, occupation), date of the visit to the cave, identification of any accompanying person, time spent inside the cave, if they came close to the fence (near the bat population), if they observed presence of livestock and of animal material inside the cave (if yes, whether they had been in the proximity), use of protective face masks inside the cave, any other general comments about the visit, as well as frequency of contact with livestock. Health-related data such as day of onset and type of symptoms, date of visit to the primary healthcare centre or hospital and dates of blood sampling were later compiled.

#### Detection of Q fever in humans

Paired blood samples from suspected cases were taken at the hospitals or healthcare centres separated by 2–3 weeks. The results were reported by the microbiology services to the Epidemiological Surveillance Unit, which had access to the medical records and followed up the results of the serological testing. Some cases, especially those patients admitted to the hospital, were also tested for other bacteria causing atypical pneumonia like *Legionella* and *Mycoplasma,* in addition to *C. burnetii*.

Serological analyses were performed at different hospitals and healthcare services using a commercial indirect immunofluorescence antibody test (IFAT, most frequently with the I + II IFA IgG/IgM/IgA assay, Vircell, Granada, Spain). Serological results from patients who seroconverted, i.e. a fourfold rise in *C. burnetii* phase II IgG or IgM antibody titres in two blood samples collected 2–3 weeks apart, were considered positive. Laboratory results were considered inconclusive when only one blood sample was available and IFAT titres for phase II IgG were greater than 1:256 or 1:320, depending on the assay used.

#### Case definition

A confirmed case was defined as a person who had visited the cave between December 2020 and October 2021 and showed compatible clinical symptoms (fever, pneumonia and/or hepatitis) with positive results in the first determination of phase II IgG or IgM antibodies or seroconversion (always with phase II IgM positive) within 40 days after the visit. Probable cases were visitors with the aforementioned clinical symptoms but without confirmation by laboratory analyses or visitors reporting milder respiratory symptoms with positive IgM.

### Investigations of goat and sheep farms

Veterinarians of the competent authorities of Animal Health in Bizkaia handled the investigation of animals. In February 2021, blood samples were collected from one sheep and six goat farms that grazed in the vicinity of the cave (< 1.6 km). Between October 2021 and January 2022, blood samples were taken from another 70 farms located within a distance of 7 km from the cave (Figure 1). On 55 of these 70 farms, dust was also collected from various surfaces within the animal premises using sterile cotton swabs. On six additional farms in the same area, a dust sample but no blood samples were taken. Thus, at a total of 76 farms, blood and/or dust samples were taken for serological and molecular testing, respectively. No other samples such as uterine fluids, faeces or milk were collected from the animals.

### Environmental sample collection inside the cave

Environmental samples were taken inside the cave at 10 different time points (March 2021–February 2023), and included faecal droppings, dust, and/or aerosol samples from four sections of the cave (Figure 1C, yellow circular shadows). Faecal samples (only collected in March 2021) consisted of six composite samples of 10–20 g of old, dry and hard faecal droppings that based on their shape and size were most likely of caprine origin.

Aerosol samples were collected in a gelatine filter adapted to a portable air sampler (MD8 Airport, Sartorius, Goettingen, Germany) performing 10 min aspirations at 50 L/min in each sampling section. Dust samples were collected from the rock cavities in the walls and the surface of the fence using sterile cotton swabs. Dust samples were taken from 10 sites along the four sections of the cave and the swabs were processed for DNA extraction. In addition, 2–3 g of dust was collected from the area surrounding the fence separating the gallery that hosted the bats and used for viability studies of *C. burnetii*.

### *Coxiella burnetii* detection on farms by serological and molecular methods

Serological analyses of blood samples from goats and sheep were performed using a commercial ELISA test (CHEKIT Q Fever Antibody ELISA kit, IDEXX, Liebefeld-Bern, Switzerland). Differences in seropositivity (at the farm and animal level) among herd types (goat, sheep or mixed) were evaluated using Fisher’s exact test or chi-squared test (R statistical software, version 3.6.1 [16]).

For molecular testing of environmental samples, we extracted DNA using a commercial kit (NZY Tissue gDNA Isolation kit, NZYTech, Lisbon, Portugal) directly from faecal and dust samples, and after a pre-treatment step for aerosol samples, as described elsewhere [17]. After DNA extraction, detection of *C. burnetii* DNA was performed by real-time PCR amplification of the transposon-like repetitive region *IS1111* of *C. burnetii*, as previously described [18]. Samples with real-time PCR quantification cycle (Cq) values below 35 were considered positive, weakly positive if Cq is between 35–40, and negative if Cq is above 40.

### Viability of *Coxiella burnetii*

Testing for viability of *C. burnetii* spores was done by culturing in Vero cells (African green monkey epithelial cells VERO C1008, Vero 76, clone E6, Vero E6 ATCC CRL-1586) in biosafety level 3 (BSL3) facilities. A dust sample of 100 mg was homogenised in 600 µL of cell culture medium and centrifuged (200 × g, 2 min). An aliquot of 100 µL of the supernatant was directly inoculated into shell vials (SV). Thereafter, the number of *C. burnetii* genome equivalents present in each inoculum was determined by quantitative real-time PCR (qPCR) using 5 µL of DNA (in triplicate) and specific primers and probe targeting the *com1* gene, as described [19]. In each qPCR run, a standard curve was generated using 10-fold serial dilutions of a known concentration of Nine Mile (RSA439) phase II strain of *C. burnetii* DNA. A broad-spectrum antibiotic-antifungal cocktail containing 10,000 units/mL of penicillin (Life Technologies Limited, Gibco, Paisley, UK), 10,000 µg/mL of streptomycin (Life Technologies Limited, Gibco, Paisley, UK), 4,000 µg/mL of gentamicin (Fisher BioReagents™, Geel, Belgium) and 25 µg/mL of amphotericin B (Life Technologies Corporation, Gibco, Grand Island, New York, USA) was added to the SV culture media to avoid microbial contamination [20]. Cell cultures were incubated at 37°C with 5% CO_2_. On Day 6 post-inoculation, 600 µL of the *C. burnetii* cell culture was harvested from the SV and transferred into T25 culture flasks containing a Vero layer. Then, two more passages of 1,000 µL of harvested cells were performed at weekly intervals [21]. At Day 6 post-inoculation and before each passage, 200 µL of the cell culture were collected for DNA extraction and qPCR following the procedure described above. To assess viability, molecular quantitation of *C. burnetii* in cells harvested during the second and/or third passages (A) was compared with the inoculated amounts (A’); if A − A’ > 0.5 log genome equivalents/mL, *Coxiella* was considered viable.

### *Coxiella burnetii* genotyping

Dust samples from the farms and the cave environment that tested positive in real-time PCR with Cq below 31 were genotyped by a 10-loci single nucleotide polymorphism (SNP) discrimination test using real-time PCR as described elsewhere [22]. Briefly, 10 real-time PCR reactions were performed per dust DNA sample, each including two primers and two MGB TaqMan probes (labelled with VIC and FAM at the 5’ end, respectively) (Life Technologies S.A., Alcobendas, Spain) to detect point mutations at each of the 10 sites. *Coxiella burnetii* Nine Mile strain was used as a positive control.

## Results

### Epidemiological investigation

A total of 132 cases were investigated, including nine rock climbers, 108 visitors and 15 staff (14 cleaning and disinfection team members and one police officer). Of these, 108 met the case definition for confirmed (n = 88) or probable cases (n = 20) with 49.1% (n = 53) showing seroconversion (Table 1). Sixty-nine (63.9%) of the cases were men and the median age was 42 years (range: 1–68); 17 cases were aged 14 years and younger. Pneumonia was the most common diagnosis (n = 53; 49.1%) followed by febrile syndrome (n = 45; 41.7%); 27 patients required hospitalisation, but no fatalities occurred. Hepatitis was not diagnosed.

**Table 1:** Characteristics and clinical presentation of cases of the Q fever outbreak, Bizkaia, Spain, December 2020–October 2021 (n = 108 cases)

Epidemiological data	Group investigated						Total (n = 132)	
	Climbers (n = 9)		Visitors (n = 108)		Staff^a^ (n = 15)			
Cases (n)								
Confirmed	5		81		2		88	
Probable	1		16		3		20	
Total	6		97		5		108	
Visit date to the cave	Dec 2020–Jan 2021		Mar 2021–Apr 2021		May 2021–Oct 2021		Dec 2020–Oct 2021	
Median age in years (range)								
Males	37 (29–48)		40 (1–68)		43 (22–58)		42 (1–68)	
Females	NA		40 (6–61)		31 (31)		39 (6–61)	
	n	%	n	%	n	%	n	%
Sex								
Males	6	100	59	60.8	4	80.0	69	63.9
Females	0	0	38	39.2	1	20.0	39	36.1
Clinical presentation								
Pneumonia	5	83.3	48	49.5	0	0	53	49.0
Fever	1	16.7	42	43.3	2	40.0	45	41.7
Asymptomatic	0	0	7	7	3	60	10	9
Seroconversion	4	66.7	48	49.5	1	20.0	53	49.1
Hospitalisation	4	66.7	23	23.7	0	0.0	27	25.0

^a^ Cleaning and disinfection team and a regional police officer.

NA: not applicable.

**Figure 2:**
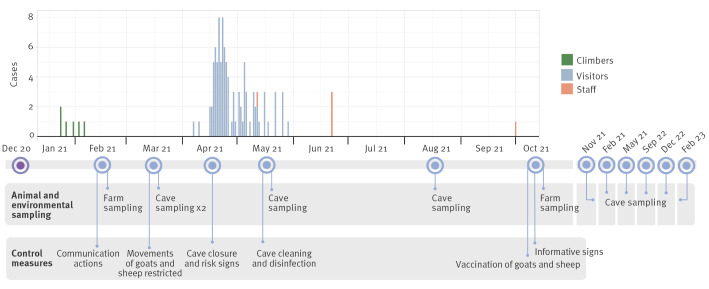
Timeline of case notifications, animal and environmental sampling, and control measures taken in a Q fever outbreak, Bizkaia, Spain, December 2020–October 2021 (n = 108 cases)

As shown in the epidemic curve (Figure 2), the first cases were rock climbers who visited the cave between December 2020 and January 2021. Subsequent cases visited the cave between March and April, most of them (n = 97; 74%) during the Easter holidays (first 2 weeks of April 2021). The last reported cases were among cleaning and disinfection workers inside the cave in May 2021 after it was closed to the public, and in October 2021, an individual guarding the cave access.

According to the questionnaire, even though the use of face protection masks (indoors and outdoors) was recommended to the general population at the time because of the COVID-19 pandemic, only half of the affected visitors reported wearing a mask and a third of them reported inconsistent use during the cave visit. About 80% (n = 86) of the visitors had approached the fence where the bats resided. The respondents also mentioned an abundance of circulating dust inside the cave. The cleaning and disinfection team was specifically requested to wear personal protective equipment, but some persons reported having occasionally removed the face protection mask during breaks, including for drinking and eating, in an area not far from the cave.

Of note, in the questionnaire, one of the first cases, a climber who visited the cave with his dog in January 2021 reported the presence of newborn kids and a placenta. He recalled his dog playing with and biting the placenta, which the climber then threw over the fence near the bat population.

### Investigation of animal and environmental samples

In February 2021, animals at seven nearby goat and sheep farms were investigated to search for possible animal sources of Q fever infections. Samples from 31 of the 117 (26.5%) animals tested were serologically positive (all goats) (Table 2). Of the 1,261 animals (361 goats and 900 sheep) from the 70 farms tested between October 2021 and January 2022, 4.8% were serologically positive (Table 2). There were no significant differences in seropositivity between farm types although caprine samples were slightly more often seropositive compared with ovine samples.

**Table 2:** Serological results of Q fever in goat and sheep farms, Bizkaia, Spain, March 2021–January 2022 (n = 1,378 samples)

Sampling date	Animal species	Farms tested		Animals tested		
		Number	Positive	Number	Positive	%
Feb 2021	Total	7	4	117	31	26.5
	Goat	6	4	98	31	31.6
	Sheep	1	0	19	0	0
Oct 2021–Jan 2022	Total	70	22	1,261	61	4.8
	Goats	24	6	222	14	6.3
	Sheep	36	10	609	29	4.8
	Mixed (goats and sheep)ª	10	6	430	18	4.2

ª Small-scale non-professional farms with a mix of goats and sheep.

In 44 of the 61 (72.1%) farms where environmental dust samples were taken, a positive or weakly positive PCR result was obtained. *Coxiella burnetii* DNA was more often detected from dust samples from sheep and mixed farms (20/28 and 8/10, respectively) than from goat farms (16/23), but the differences were not significant.

Environmental samples collected from the cave on 4 March 2021 provided preliminary results pointing to *C. burnetii* as the cause of the human infections. *Coxiella burnetii* DNA was detected by real-time PCR in composite faecal, dust and aerosol samples. Four of the six faecal samples collected inside the cave in March were PCR-positive for *C. burnetii* (Table 3). The samples collected by the main entrance of the cave (F1–F3) and by the fence (F4) were positive, whereas samples taken further inside the cave (F5) and at the alternative entrance (F6) were negative (Figure 1). Therefore, *C. burnetii* was found to be more concentrated in areas where faecal droppings were more abundant (Sites F1–F4) compared with the innermost galleries (Sites F5–F6).

**Table 3:** Real-time PCR amplification of *Coxiella burnetii* from environmental samples collected inside a cave, Bizkaia, Spain, March 2021–Feb 2023 (n = 122 samples)

Sample type and sampling site^a^	Cq values^b^									
	4 Mar 2021	22 Mar 2021	18 May 2021	2 Aug 2021	2 Nov 2021	28 Feb 2022	9 May 2022	22 Sep 2022	19 Dec 2022	2 Feb 2023
Faeces										
Main entrance (F1)	31.3	ND	ND	ND	ND	ND	ND	ND	ND	ND
Main entrance (F2)	35.7									
Main entrance (F3)	34.3									
Fence (F4)	32.3									
Interior (F5)	Und.									
Alternative entrance (F6)	Und.									
Dust^c^										
Main entrance (D1)	31.1	ND	34.6	36.9	35.1	34.7	Und.	35.5	36.6	ND
Main entrance (D2)	31.3		31.3	26.9	31.8	27,0	Und.	32.1	35.9	
Fence (D3)	**28.7**		**25.6**	**24.9**	**28.6**	**22.5**	**29.1**	**27.7**	**30.0**	
Interior 1 (D4)	31.9		33.6	34.2	35.8	34.2	34.3	34.6	Und.	
Interior 2 (D5)	32.5		35.4	36.7	Und.	33.3	34.6	Und.	Und.	
Interior 3 (D6)	Und.		33.7	Und.	36.1	Und.	37.3	Und.	Und.	
Interior 4 (D7)	Und.		34.2	Und.	Und.	35.1	36.1	36.8	Und.	
Interior 5 (D8)	Und.		Und.	Und.	Und.	34.4	34.6	Und.	Und.	
Alternative entrance (D9)	35.4		Und.	Und.	Und.	35.9	34.9	Und.	Und.	
Alternative entrance (D10)	Und.		35.0	Und.	Und.	Und.	Und.	Und.	Und.	
Aerosols										
Aerosol 1 (A1)	Und.	32.2	Und.	33.4	Und.	Und.	ND	36.3	35.7	Und
Aerosol 2 (A2)	35.4	24.6	26.0	Und.	27.8	29.2		25.3	24.9	25.9
Aerosol 3 (A3)	Und.	Und.	36.3	Und.	Und.	Und.		33.3	36.8	29.6
Aerosol 4 (A4)	33.2	Und.	Und.	Und.	Und.	Und.		36.4	Und.	Und.
Wind direction at sampling^d^	South-East	North	South-West	North	South-West	South-East	North-East	North	South-East	North-West

Cq: quantification cycle; ND: not done; Und.: undetermined (negative).

^a^ See Figure 1 for location of sampling sites inside the cave.

^b^ Cq interpretation as follows: Cq < 35: positive; Cq > 35 and ≤ 40: weakly positive; Cq > 40: negative. The lowest Cq value (higher bacterial load) obtained in dust during each sampling session is marked in bold. PCR target was the transposon-like repetitive region *IS1111*.

^c^ On 22 March 2021 and 2 February 2023, dust samples were taken only for viability studies.

^d^ Data were provided by the Basque Agency of Meteorology (Euskalmet, https://www.euskalmet.euskadi.eus/inicio) and collected at a meteorological station located 6.8 km from the cave.

*Coxiella burnetii* was detected in dust (47/80) and aerosol (18/36) samples taken from March 2021 to February 2023 (Table 3). Findings of *C*. *burnetii* in the environmental dust samples concentrated to the first two sections of the cave, the areas where animals seemed to stay or rest frequently. The sampling site that consistently had the lowest Cq value (highest bacterial load) throughout the study period was the fence (D3, A2). Samples from the fence (D3) were always positive; dust and aerosol samples from all the other sampling sites were positive at least once. In the last sampling of dust (December 2022), we still detected *C. burnetii* from three sites. The wind direction varied between samplings (Table 3).

### *Coxiella burnetii* genotyping and viability

In the SNP analysis of *C. burnetii* detected from five dust samples collected by the fence (D3) inside the cave in March, May, and November 2021 and February and May 2022, we identified the genotype SNP-8. When 12 dust samples from goat and sheep farms were genotyped, two genotypes were identified: SNP-8 (five goat farms and one sheep farm) and SNP-3 (three sheep, two goat and one mixed farm).

Of the nine dust samples taken by the fence (D3) between March 2021 and February 2023 and cultured on Vero cells: *C. burnetii* could be grown in six of them; one sample was contaminated and growth was not detected in two samples (Table 4). These results confirmed the presence of viable *Coxiella* inside the cave from the beginning of the outbreak until the sampling in December 2022.

**Table 4:** Culture of *Coxiella burnetii* on Vero cell lines from dust sample homogenates collected inside a cave, Bizkaia, Spain, March 2021–February 2023 (n = 9)

Sampling date	Inoculated amount (GE/mL)	Culture on Vero E6 cell lines (GE/mL)				
		Day 6 p.i.	Passage 1	Passage 2	Passage 3	Viable *C. burnetii*
Mar 2021	3.68 × 10^4^	7.39 × 10^4^	4.27 × 10^5^	5.17 × 10^5^	2.63 × 10^6^	Yes
May 2021	3.56 × 10^4^	8.56 × 10^3^	Contaminated	Not performed		
Aug 2021	4.67 × 10^7^	8.17 × 10^7^	1.31 × 10^8^	1.29 × 10^8^	4.76 × 10^7^	Yes
Nov 2021	2.43 × 10^7^	2.54 × 10^7^	5.73 × 10^7^	5.05 × 10^7^	9.09 × 10^7^	Yes
Feb 2022	3.96 × 10^6^	4.91 × 10^6^	1.35 × 10^7^	1.27 × 10^7^	1.94 × 10^7^	Yes
May 2022	4.88 × 10^5^	8.24 × 10^5^	1.82 × 10^6^	2.98 × 10^6^	4.71 × 10^6^	Yes
Sep 2022	1.78 × 10^6^	2.46 × 10^6^	2.94 × 10^6^	8.59 × 10^5^	1.27 × 10^6^	No
Dec 2022	5.33 × 10^5^	1.61 × 10^6^	1.04 × 10^6^	1.15 × 10^6^	0	Yes
Feb 2023	6.47 × 10^5^	8.65 × 10^5^	2.04 × 10^5^	3.45 × 10^5^	0	No

GE: genome equivalents of *C. burnetii* determined by quantitative real-time PCR targeting the *com1* gene; p.i.: post-inoculation.

### Outbreak control measures

The timeline of the control measures taken is displayed in Figure 2. Once the outbreak was identified in February 2021, we informed healthcare workers to identify as many infections as possible and prevent further infections. Q fever is well-known among physicians of the Basque Country and annual rates are among the highest in Spain [12]. Still, when the first cases were detected, an informative note was sent to the local healthcare network to increase awareness of possible infections among individuals with compatible symptoms and who had visited the cave. Also, the outpatient healthcare centre in the municipality where the cave is located was asked to be on the alert for any cases with Q fever-compatible symptoms even for others who did not visit the cave. Simultaneously, the Basque Mountain Federation was contacted and asked to inform all the members about the outbreak and recommend them to visit their healthcare centre in case of symptoms compatible with the disease. Likewise, a press release was published to inform the public and ask cave visitors to report any illnesses with compatible symptoms and seek medical care. Vaccination of humans was not among the measures taken since it is not in use in Spain.

On 29 April 2021, the Deputy Director of Public Health of Bizkaia requested to close the cave for visitors and to secure the entrance with an electric fence. In May 2021, the cave was cleaned and disinfected by first soaking the faecal material on the floor with Virkon disinfectant (Bayer Hispania S.L.), removing the faeces and repeating the disinfection treatment. The area surrounding the fence that separates the gallery hosting the bats was not disinfected because of the proximity to the bats. In October 2021, information signs about the risk of Q fever infection were placed in the surroundings of the cave to prevent entry.

Movement of goats and sheep from farms in the area was restricted for several months after the parturition season (March–July 2021). In October 2021, goat and sheep herds were vaccinated (Coxevac, CEVA Animal Health, Santé Animale, Libourne, France). This vaccination programme will involve several phases that will be monitored over the coming years.

On 19 May 2023, when the numbers of viable *Coxiella* in the cave environment decreased to negligible levels and two months had passed since the end of the parturition season for the goats and sheep, the cave was re-opened to the public.

## Discussion

This large Q fever outbreak in Spain occurred during the COVID-19 pandemic, when restrictions limited movements to nearby localities and outdoor leisure activities were preferred. Hence, during the Easter holidays, the cave attracted many local visitors who considered it an open natural environment and disregarded pandemic recommendations to use a face mask (also outdoors) and were consequently more exposed to *C. burnetii*. Pneumonia, the main clinical presentation of Q fever in northern Spain [12], was also the most common clinical presentation in this outbreak. Therefore, upon arrival at the hospital or primary healthcare centre, patients were first tested for severe acute respiratory syndrome coronavirus 2 (SARS-CoV-2), which delayed diagnosis and confirmation of the first Q fever cases. In addition, patients with respiratory symptoms who tested positive for SARS-CoV-2 were no longer investigated for Q fever. These reasons, and the fact that not all visitors to the cave were tested, suggests that the true number of Q fever cases could have been higher than reported.

Human Q fever outbreaks are primarily associated with goats and sheep [1,23,24]. In Bizkaia, the province where the cave is located, goats have been involved in all Q fever outbreaks reported in the past 10 years whenever the source was identified [15]. Also, in the outbreak we describe, goats were the most likely source of the environmental contamination of the cave. The presence of goat parturition remains inside the cave as well as goats in the surrounding area, days before the symptom onset of cases, were reported by the rock climbers during the epidemiological investigation. The *C. burnetii* genotype (SNP-8) identified in the environmental samples collected inside the cave is the most commonly detected genotype in goats in the municipality and one of the genotypes previously found in human cases in Q fever outbreaks reported in the Basque Country [15]. Unfortunately, no blood samples from the cases were analysed by PCR, thus preventing genotyping and the possibility to compare isolates from humans and the environment. The other genotype (SNP-3) found in dust samples of some of the ovine and caprine farms had previously been detected in a white stork in the region [25] but not in small ruminants [15]. Future studies may provide further evidence of the importance of this genotype in public and animal health.

*Coxiella burnetii* infection in goats can cause high abortion rates. Infected animals shed millions of bacteria via faeces, fluids, placentas and aborted foetuses [26], thus contaminating the environment via aerosols. The movement of the animals and the wind likely contributed to the spread of the contamination in the cave, and the detection of *C. burnetii*-contaminated aerosols in the environment clearly confirmed the risk of infection for susceptible people or animals [6,21,27]. Here, infected parturition materials and faeces most likely contaminated the ground of the caves, while dry periods enhanced the formation and propagation of infectious dust and aerosols. The first evidence came from samples collected during the first visit to the cave on 4 March 2021 when *C. burnetii* DNA was detected by real-time PCR in composite faecal samples, dust, and aerosols collected inside the cave. This and previous studies showed the usefulness of environmental sampling to successfully investigate Q fever outbreaks [28-30].

*Coxiella burnetii* endospores are extremely resistant to heat, pressure and desiccation and can remain viable for several months under conditions of high humidity, low temperatures and absence of sunlight [1]. Here, we successfully used Vero cell cultures for viability studies without the need to use mouse inoculation tests, as in other studies [21]. Although the cave was closed for both visitors and domestic animals soon after the outbreak was declared, cell culture results indicated that *C. burnetii* remained viable inside the cave for 24 months (December 2020–December 2022). Bats and small mammals can be infected with *C. burnetii* [31,32], but their role in shedding or maintaining viable *Coxiella* in the environment is unknown. Unfortunately, neither the bats that inhabit the cave nor the small mammals present in the vicinity could be tested to assess their possible role in the infection cycle inside the cave.

Effective control and prevention of Q fever in humans requires the identification of *C. burnetii* infection in domestic ruminants. Although infected goats were the most likely origin of the outbreak, *C. burnetii* shedding by animals was only tested at farm level (environmental dust) and not at animal level through examination of vaginal swabs, milk or faeces. This hampered the identification of farms with potentially shedding animals and, thus, the possible source of the outbreak. When *C. burnetii* infection is suspected or detected in a herd, prevention efforts should focus on reducing animal infection and environmental contamination. Therefore, the vaccination programme designed at the municipality level is expected to significantly reduce *C. burnetii* shedding in goats and sheep in future parturitions.

This study showed that Q fever outbreaks can occur in unexpected places, such as natural areas visited for sport or recreation activities, where the presence of animals is common. Several implications for public health can be conveyed from this investigation. Firstly, the general public must be aware of the potential risk of zoonotic infections from animals, not only through direct contact but also when sharing natural spaces. Our study confirms that *C. burnetii* transmission from the environment to humans can occur several months after environmental contamination by infected animals. Therefore, citizens should follow recommendations by public health experts. Secondly, there is a high occupational risk of infection for persons involved in outbreak control measures, as reported elsewhere [33]. Thus, proper use of personal protective equipment among these workers should be emphasised. Thirdly, implementing surveillance and control programmes to reduce the prevalence of Q fever in domestic ruminants (the main source of human infection) is key to reduce the risk of infections in humans, particularly in endemic areas with a high prevalence in domestic ruminants. However, when these measures fail and outbreaks do occur, a comprehensive One Health approach considering human, animal and environmental factors should be followed for a successful outbreak investigation. Therefore, clinicians should be aware of the importance of obtaining appropriate human samples from suspected Q fever cases at the time of bacteraemia for *C. burnetii* molecular characterisation to be able to compare genotypes from human, animal, and environmental sources.

## Conclusions

The comprehensive cross-sectoral One Health approach and the public health measures adopted proved to be effective to control this Q fever outbreak, since no new human cases have been detected since October 2021. Limitation of access to the cave for goats and sheep, as well as the cleaning measures, resulted in a decrease of viable *C. burnetii* spores in the cave environment. This, along with the Q fever control programme in goats and sheep including the established vaccination plan, should contribute to reduce *C. burnetii* environmental contamination in the area. However, in endemic regions, Q fever outbreaks can occur in unexpected places, such as natural sites for sport or recreation where animals are present. Therefore, strengthening alertness and preparedness as well as implementing robust surveillance and response capacity are keys to limit transmission.
